# Long Interspersed Nuclear Element-1 Hypomethylation and Oxidative Stress: Correlation and Bladder Cancer Diagnostic Potential

**DOI:** 10.1371/journal.pone.0037009

**Published:** 2012-05-15

**Authors:** Maturada Patchsung, Chanchai Boonla, Passakorn Amnattrakul, Thasinas Dissayabutra, Apiwat Mutirangura, Piyaratana Tosukhowong

**Affiliations:** 1 Department of Biochemistry, Faculty of Medicine, Chulalongkorn University, Bangkok, Thailand; 2 Department of Surgery, Faculty of Medicine, Chulalongkorn University, Bangkok, Thailand; 3 Department of Anatomy, Faculty of Medicine, Chulalongkorn University, Bangkok, Thailand; The Chinese University of Hong Kong, Hong Kong

## Abstract

Although, increased oxidative stress and hypomethylation of long interspersed nuclear element-1 (LINE-1) associate with bladder cancer (BCa) development, the relationship between these alterations is unknown. We evaluated the oxidative stress and hypomethylation of the LINE-1 in 61 BCa patients and 45 normal individuals. To measure the methylation levels and to differentiate the LINE-1 loci into hypermethylated, partially methylated and hypomethylated, peripheral blood cells, urinary exfoliated cells and cancerous tissues were evaluated by combined bisulfite restriction analysis PCR. The urinary total antioxidant status (TAS) and plasma protein carbonyl content were determined. The LINE-1 methylation levels and patterns, especially hypomethylated loci, in the blood and urine cells of the BCa patients were different from the levels and patterns in the healthy controls. The urinary TAS was decreased, whereas the plasma protein carbonyl content was increased in the BCa patients relative to the controls. A positive correlation between the methylation of LINE-1 in the blood-derived DNA and urinary TAS was found in both the BCa and control groups. The urinary hypomethylated LINE-1 loci and the plasma protein carbonyl content provided the best diagnostic potential for BCa prediction. Based on post-diagnostic samples, the combination test improved the diagnostic power to a sensitivity of 96% and a specificity of 96%. In conclusion, decreased LINE-1 methylation is associated with increased oxidative stress both in healthy and BCa subjects across the various tissue types, implying a dose-response association. Increases in the LINE-1 hypomethylation levels and the number of hypomethylated loci in both the blood- and urine-derived cells and increase in the oxidative stress were found in the BCa patients. The combination test of the urinary hypomethylated LINE-1 loci and the plasma protein carbonyl content may be useful for BCa screening and monitoring of treatment.

## Introduction

Carcinogenesis of the urinary bladder is complex because both genetic mutations and epigenetic alterations play important roles. Furthermore, inflammation and oxidative stress critically contribute to development of bladder cancer (BCa) [1]–[3]. Various lines of evidence report an increased oxidative stress in patients with BCa [3]–[6]. Oxidative stress is a condition of the excessive production of reactive oxygen species (ROS) and/or a reduction of antioxidants. ROS directly damages the cellular DNA and promotes tumor development not only through genetic mutations but also through epigenetic alterations [7]. Common epigenetic alterations in human cancers include global hypomethylation and regional (site-specific CpG island promoter) hypermethylation of the tumor suppressor genes [8], [9]. Hypomethylation of the cancer genome occurs on the repetitive sequences and retrotransposable elements [10], which accelerates the genomic instability [10]–[13] and alters gene expression [14]. The best characterized and most abundant retrotransposon in the mammalian genome is the long interspersed nuclear element-1 (LINE-1 or L1), and it is known that LINE-1 hypomethylation is associated with many malignancies [15], [16].

In urothelial cancer, the hypomethylation of LINE-1 was first demonstrated in urothelial cell lines and tumor tissues by Schulz and colleagues based on experiments using methylation-sensitive restriction enzymes (*Hpa*II/*Msp*I) and Southern blotting [17], [18]. The LINE-1 hypomethylation corresponded well with an increase in the LINE-1 transcripts and a decrease in gene containing LINE-1 mRNA [14]. Later, our group employed a combined bisulfite restriction analysis (COBRA) PCR to demonstrate LINE-1 hypomethylation in various carcinomas tissues including bladder cancer [15]. Choi et al. used bisulfite-PCR pyrosequencing and showed an obvious hypomethylation of LINE-1 in bladder tumor tissues relative to the adjacent normal tissues [19]. The reduction of 5-methylcytosine levels in leukocyte DNA was shown in Spanish patients with bladder cancer relative to the matched controls [20]. Recently, the hypomethylation of LINE-1 in peripheral blood cells was associated with an increased risk for bladder cancer, especially in women [21]. Among nonsmoking Chinese, similar result that LINE-1 hypomethylation in lymphocytes was associated with increased risk for bladder cancer was demonstrated [22]. Previously, the methylation of LINE-1 in urinary exfoliated cells and its implication in bladder cancer has not been investigated. Even though LINE-1 hypomethylation and increased oxidative stress are well recognized in BCa patients, the association between these two phenomena has not been explored.

**Figure 1:**
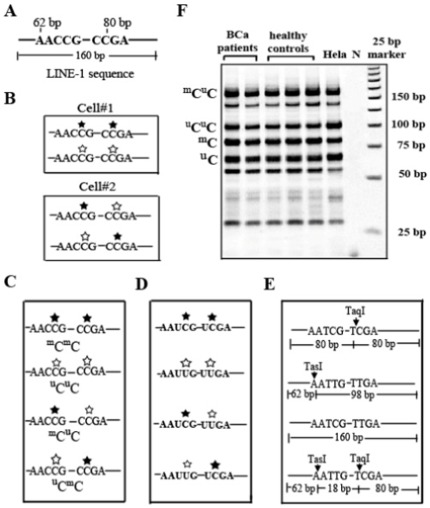
The methylation patterns of LINE-1 detected by COBRA PCR. A: The detecting 5′ UTR of the LINE-1 sequence contains two CpG dinucleotide loci, and the PCR amplicon size of LINE-1 is 160 bp. B: There are 4 possible methylation patterns of the LINE-1 sequence (one cell contains two alleles). The solid stars represent methylated cytosines and the hollow stars represent unmethylated cytosines. C: COBRA LINE-1 separated the detecting region into four products: ^m^C^m^C, ^u^C^u^C, ^m^C^u^C and ^u^C^m^C. D: After the bisulfite treatment, the unmethylatated cytosine residues are converted to uracil, but the methylated cytosine residues are unaltered. This leads to retention or loss of CpG containing restriction enzyme sites, respectively. E: The PCR products are digested with TaqI (recognition sequence: TCGA) and TasI (recognition sequence: AATT) restriction enzymes. A TaqI positive digest yields two 80-bp DNA fragments, while a TasI positive digest yields a 62- and a 98-bp fragment. F: Representative gel image for COBRA LINE-1 assay. Lanes 1–2: DNAs from BCa patients, Lanes 3–5: DNAs from healthy controls, Lane 6: DNA from HeLa cells as positive control and normalizing sample, Lane 7: negative (N) control, Lane 8: 25 bp markers.

To date, the most commonly used techniques for measuring LINE-1 methylation level are pyrosequencing and COBRA PCR. These two methods had have similar efficacy in the detection of methylation levels and have limited margins of error [23]. The pyrosequencing of LINE-1 detects a few more CpG dinucleotides, usually three CpGs, [24] than the COBRA PCR detects (usually two CpGs). However, the LINE-1 methylation of each locus is not homogenous [23], [25], which can influence expression and stability of the genome in cis [16]. Consequently, the LINE-1 methylation levels do not precisely represent the biological roles of the epigenomic modification [16]. To improve LINE-1 methylation evaluation, in addition to overall methylation level, we used COBRA PCR of LINE-1 to classify the genome-wide LINE-1 loci into four groups, ^m^C^m^C (hypermethylated), ^u^C^u^C (hypomethylated), ^m^C^u^C and ^u^C^m^C (partial methylated), and calculate the percentages of each group (Fig. 1) [26]. Of note, certain LINE-1 methylation assays have a positive methylation control base which can be use to determine the efficacy of the bisulfite treatment.

To evaluate the association between oxidative stress and LINE-1 hypomethylation, we determined the methylation levels of LINE-1 in the blood and urine cells obtained from the BCa patients and from the healthy individuals. Oxidative stress, indicated by the urinary total antioxidant status (TAS) and plasma protein carbonyl content, was compared between the two groups. The association of the LINE-1 methylation levels with the oxidative stress status was evaluated, and the usefulness of LINE-1 hypomethylation detection in the urinary exfoliated cells and the overall oxidative stress as markers for BCa diagnosis was assessed.

## Materials and Methods

### Participants

Sixty-one patients with BCa that were admitted to the King Chulalongkorn Memorial Hospital, Bangkok, Thailand between 2009 and 2010 were recruited for the study. All of the patients had histological proof of a superficial transitional cell carcinoma. Any BCa patients that presented with a muscle invasive phenotype were excluded from this study. Age- and gender-matched healthy individuals (n = 45) were used as controls. The controls were selected from members of the healthy elderly community at Lumpini Park, Bangkok, where they came everyday to do exercise. The healthy condition was confirmed by direct interview and their annual medical check-up to ensure that there was no history of urinary disorders and any malignancies. All subjects voluntarily participated in the study. The study protocol was reviewed and approved by the Ethics Committee of the Faculty of Medicine, Chulalongkorn University, Bangkok, Thailand. Written informed consents were obtained from all participants.

### Specimen collection and DNA extraction

Heparinized blood and midstream spot morning urine (between 8:00 am and 12:00 pm) were collected from all participants. The blood samples were centrifuged to collect the plasma and buffy coat. The plasma samples were kept at −80°C until testing. The buffy coat samples were immediately subjected to DNA extraction. To isolate the urinary exfoliated cells, 50 ml of the urine samples was centrifuged at 4,000 rpm for 20 min at 4°C. The cell pellet was collected and stored in RNA stabilizer (QIAGEN, Germany) at −80°C until analysis. Of the 61 BCa patients, 14 had available cancerous tissues, which were obtained during the operation of transurethral resection, immediately stored in RNA stabilizer and kept at −80°C for the LINE-1 measurement. Due to low amount of nucleated cells in urines, especially in healthy urines, DNAs from urinary exfoliated cells were successfully extracted from 30 BCa patients and 14 healthy controls. The DNA was extracted from peripheral blood buffy coats, urinary exfoliated cells and cancerous tissues using the High Pure PCR Template Preparation Kit (Roche Diagnostics, Indianapolis, USA). The incomplete specimen collection was considered as a weakness of the study. In BCA group, the blood, urinary exfoliated cells and cancerous tissues were accounted for 61 (100%), 30 (49.18%) and 14 (22.95%), respectively. Fourteen BCa patients (22.95%) had all types of specimens for analysis. In healthy control group, 45 (100%) blood and 14 (31.11%) urinary exfoliated cells samples were analyzed. Fourteen control subjects (31.11%) had both specimens for analysis. The power to detect the correlation observed for an n = 14 was calculated using the G*Power 3.1.3 software [27], and the output displayed the power of 0.66 (inputs: two-tails, effect size = 0.56, α = 0.05, total sample size = 14).

### LINE-1 methylation by COBRA PCR

In brief, genomic DNAs (250 ng) derived from blood, urine and cancerous tissue samples were treated with sodium bisulfite as previously described [15]. The bisulfite-treated DNA was subjected to 35 cycles of PCR with LINE-1-F (5′-CCGT AAG GGGTTAGGGAGTTTTT-3′) and LINE-1-R (5′-RTAAAACCCTCCRAACCAAAT ATAAA-3′) primers with an annealing temperature of 50°C to generate 160 bp PCR amplicons. The amplicons were further digested with *Taq*I (2 U) (sticky end) and *Tas*I (2 U) (sticky end) in NEB3 buffer (MBI Fermentas, Glen Burnie, MD) at 65°C overnight. The digested products were then electrophoresed in an 8% nondenaturing polyacrylamide gel and subsequently stained with SYBR green. The experiment was performed in duplicate.

The intensities of the COBRA LINE-1 fragments in the polyacrylamide gel were quantified using a phosphoimager and ImageQuant Software (Molecular Dynamics, GE Healthcare, Slough, UK). As detected by COBRA, methylation status of the 2 CpG dinucleotides of LINE-1 loci was classified into four groups as follows: (1) LINE-1 loci containing 2 unmethylated CpGs (^u^C^u^C); (2) LINE-1 loci containing 2 methylated CpGs (^m^C^m^C); (3) LINE-1 loci containing 5′-methylated and 3′-unmethylated CpGs (^m^C^u^C); and (4) LINE-1 loci containing 5′-unmethylated and 3′-methylated CpGs (^u^C^m^C). The details for band intensity quantitation was fully described elsewhere [28]. In brief, four bands that differed in their states of methylation, including 98 bp (^u^C^u^C), 160 (^m^C^u^C ), 80 (^m^C ) and 62 (^u^C) bp, were quantified (Fig. 1). The intensity of each band was divided by its paired length as followed: 160 bp/160 (A), 98 bp/94 (B), 80 bp/79 (C) and 62 bp/62 (D). The LINE-1 methylation level (overall or total) was calculated as the percent of the methylated bands (^m^C ) intensity divided by the sum of the ^m^C and unmethylated bands (^u^C ) intensities, (C+A)/(C+A+A+B+D)×100. The percent of ^u^C^u^C (hypomethylated loci) was calculated from the followed equation: B/(((C−D+B)/2)+A+D)×100. The ^m^C^u^C (partial methylated loci) was computed from the equation: A/(((C−D+B)/2)+A+D)×100. The ^m^C^m^C (hypermethylated loci) was computed from the equation: ((C−D+B)/2)/(((C−D+B)/2)+D+A)×100. The CV of the LINE-1 methylation levels in blood, urinary exfoliated cells and cancerous tissues DNAs were 7.31% (range: 2.33–12.91%), 7.64% (range: 2.50–13.17%) and 8.00% (range: 3.56–10.54%), respectively. DNA from HeLa cells was used as a control to normalize the inter-assay methylation variation for all of the experiments (Fig. 1F). The percent of LINE-1 methylation detected in HeLa DNAs was 28.59%, and this value was used for normalization between the runs. The mean of LINE-1 methylation in HeLa DNAs was 27.74±2.33%, and the between-run CV was 8.41%.

### Total antioxidant status (TAS)

The 2, 2-diphenyl-1-picryl-hydrazyl (DPPH) reduction assay was performed to determine the TAS in urine samples. Briefly, a urine sample (20 µL) was added to of 400 µL of 10 mM phosphate buffered saline (PBS, pH 7.4) and 400 µL of freshly prepared 0.1 mM DPPH in methanol. The reaction tubes were incubated at room temperature for 20 min in the dark. The optical density (OD) at 520 nm was measured. The antioxidant capacity of the sample was calculated based on the % inhibition of DPPH radicals using the following equation: % inhibition = (OD_blank_−OD_sample_/OD_blank_)×100. L-ascorbic acid (vitamin C) was used at concentrations of 0.25, 0.5 and 1 mM to generate a standard curve (% inhibition vs. concentration). The TAS value of the urine sample was expressed as the vitamin C equivalent antioxidant capacity (VCEAC mM). All experiments were done in duplicate. The CV for urinary TAS determination was 7.81% (range: 1.02–14.59%).

### Protein carbonyl determination

The protein carbonyl content was used as an indicator of protein oxidation by ROS in plasma. A plasma sample was diluted (1∶20) with PBS (10 mM, pH 7.4) and then centrifuged at 10,000 rpm for 10 min. The plasma supernatant (250 µL) was added to 1 mL of 10 mM 2,4-dinitrophenylhydrazine (DNPH) in 2N HCl. For a reagent blank control, 1 mL of 2N HCl was added to 250 µL of the plasma supernatant. The mixtures were incubated at room temperature in the dark for 45 min and were vortexed at 10-min intervals. Then, 1.2 mL of 20% cold trichloroacetic acid was added and incubated for 10 min on ice; this was followed by centrifugation at 3,000 rpm for 15 min to collect the protein pellets. The pellets were washed three times with 2.5 mL of ethanol/ethyl acetate (1∶1, v/v) each to remove non-reacted DNPH. The washed pellets containing 2,4-dinitrophenylhydrazone were resuspended in 6 M guanidine hydrochloride/0.5 M potassium phosphate monobasic, pH 2.5, at 37°C for 15 min with vortexing. The absorbance at 375 nm was measured using the corresponding reagent blank for a zero setting. The extinction coefficient for the 2,4-dinitrophenylhydrazone at 375 nm is 22,000 M^−1^ cm^−1^ and the protein carbonyl concentration (nMol/mL) was calculated as the absorbance x 45.45 [29]. The content of protein carbonyl in the plasma was expressed per mg of total proteins (measured by the dye-binding method). The CV for determination of plasma protein carbonyl content was 8.43% (range: 2.72–13.80%).

### Statistical analysis

The data with normal distribution were presented as the mean ± standard deviation, and the data with skewed distribution were presented as median (interquartile range, IQR). For comparisons between two independent groups, the independent sample *t*-test was used for normally distributed data, and the Mann-Whitney test was used for skewed data. Pearson's correlation test was used to assess the correlation between continuous variables. A receiver operating characteristic (ROC) analysis was performed to test the ability of the COBRA LINE-1 methylation test to differentiate BCa subjects from healthy subjects. An area under the ROC curve (AUC) of 1.0 indicates perfect accuracy, whereas an AUC of 0.5 indicates that the test lacks discriminatory power. A cutoff value was selected for computing the diagnostic values. SPSS version 17.0 (SSPS Inc., Chicago, IL) and STATA version 8.0 (StataCorp, College Station, Texas) were used for all calculations. A P value<0.05 was considered statistically significant.

## Results

The BCa patients (n = 61) were between 42 and 89 years of age (65.11±12.16 years) and consisted of 52 (85.25%) males and 9 (14.75%) females. In the control group, there were 45 healthy subjects between 46 and 81 years of age (61.00±13.25 years); this group had 37 (82.22%) males and 8 (17.78%) females. Mean body mass index (BMI) of the patient and control groups were 22.24±3.71 and 23.15±2.45 (kg/m^2^), respectively. The sex distribution, age and BMI between the two groups were not significantly different.

**Figure 2:**
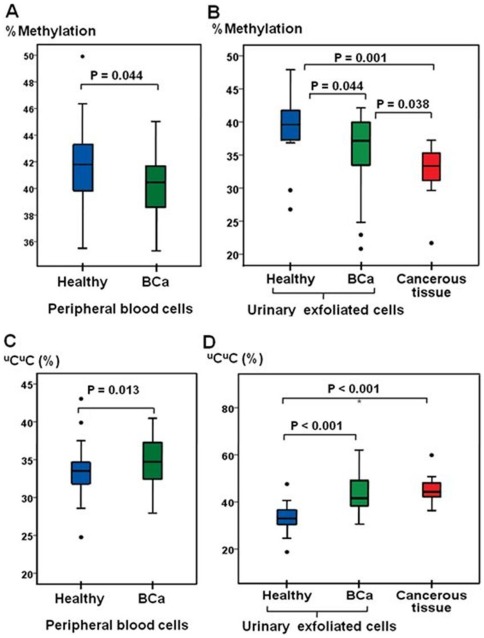
The LINE-1 methylation and ^u^C^u^C levels compared between the BCa and healthy groups. A: The percentage of LINE-1 methylation levels in the peripheral blood DNA from BCa patients (n = 61) was significantly lower than that from the healthy controls (n = 45). B: The LINE-1 methylation levels in the DNA derived from the urinary exfoliated cells from BCa patients (n = 30) were significantly decreased relative to those from the healthy controls (n = 14). The LINE-1 methylation levels in the cancerous tissues (n = 14) were significantly lower than the LINE-1 methylation levels in the urinary exfoliated cells from both BCa and healthy subjects. C: The ^u^C^u^C levels in the peripheral blood DNA from BCa patients were significantly higher than those from the healthy controls. D: The ^u^C^u^C levels in the urinary exfoliated cells of BCa patients were significantly increased compared to those from the healthy controls. The cancerous tissue ^u^C^u^C levels were not significantly different from the ^u^C^u^C levels from the BCa urinary exfoliated cells (P = 0.721), but they were significantly higher than those from the urinary exfoliated cells of healthy controls.

The methylation level of LINE-1 was measured in DNA derived from peripheral blood, urinary exfoliated cells and cancerous tissues. The percentage of LINE-1 methylation in the peripheral blood cells from the BCa patients was significantly lower than that of the healthy controls (P = 0.001) (Fig. 2A). Likewise, the LINE-1 methylation levels in the urinary exfoliated cells from the BCa patients were significantly lower than those of the healthy controls (P = 0.044) (Fig. 2B). The LINE-1 methylation levels in the BCa cancerous tissues were significantly lower than those in the urinary exfoliated cells of the BCa (P = 0.038) and healthy (P = 0.001) subjects (Fig. 2B). The percentage of ^u^C^u^C loci in the peripheral blood cells and urinary exfoliated cells of the BCa patients was significantly greater than the controls (P = 0.013 and <0.001, respectively) (Fig. 2C and 2D). The number of ^m^C^u^C loci from the urinary exfoliated cells of the BCa patients was significantly lower than that of the controls (P = 0.006), but the levels of ^m^C^u^C loci in the peripheral blood cells from the BCa and healthy groups were not different (P = 0.133) (Figure S1). These data confirmed the presence of LINE-1 hypomethylation in bladder cancer and showed similar results to those previously reported for oral cancer [26] that demonstrated the specificity of ^u^C^u^C loci to cancer DNA.

**Figure 3:**
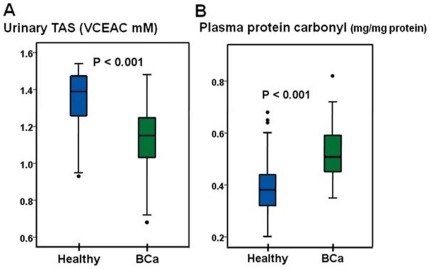
The oxidative stress biomarkers in patients with BCa. A: The level of urinary TAS in the BCa patients was significantly lower than that in the healthy controls. B: The level of the plasma protein carbonyl content in the BCa patients was significantly greater than that from the healthy controls.

The determinations of the urinary TAS and plasma protein carbonyl content were performed in both healthy and BCa groups. A significant reduction in the urinary TAS level in the BCA patients relative to the healthy controls was observed (P<0.001) (Fig. 3A). The plasma protein carbonyl content in the BCa patients was significantly higher than that in healthy controls (P<0.001) (Fig. 3B). Our findings indicated an increase in the oxidative stress in the BCa patients.

**Figure 4:**
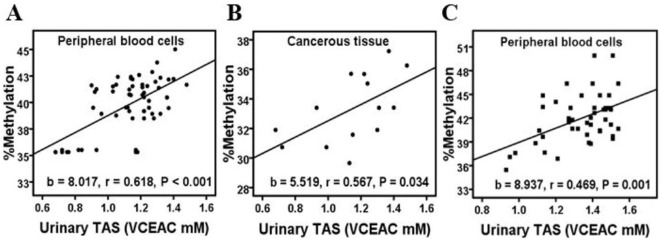
A univariate correlation analysis between the levels of urinary TAS and LINE-1 methylation. A: The urinary TAS was linearly correlated with the LINE-1 methylation levels in the peripheral blood cells of the BCa patients. B: The urinary TAS was linearly correlated with the LINE-1 methylation levels in the cancerous tissue obtained from the BCa patients. C: In the healthy group, the urinary TAS was also linearly correlated with the LINE-1 methylation levels in the peripheral blood cells. b: regression coefficient, r: Pearson's correlation coefficient. The displayed P values are from the Pearson's correlation test.

In the BCa patients, we found a strong, positive and linear correlation between the urinary TAS and LINE-1 methylation in circulating blood cells (regression coefficient = 8.017) (r = 0.618, P<0.001) (Fig. 4A). Moreover, a significant positive correlation between the urinary TAS and cancerous tissue LINE-1 methylation levels was observed (regression coefficient = 5.519) (r = 0.567, P = 0.034) (Fig. 4B). Interestingly, we also found a positive correlation between the urinary TAS and the level of LINE-1 methylation in the peripheral blood cells of the healthy individuals (regression coefficient = 8.937) (r = 0.469, P = 0.001) (Fig. 4C). The correlations between urinary TAS and the other patterns of LINE-1 methylation (^m^C^m^C, ^u^C^u^C, ^u^C^m^C and ^m^C^u^C) were evaluated (Figure S2). The significant inverse correlations between urinary TAS and the ^u^C^u^C levels in BCa peripheral blood cells (r = −0.440, P = 0.001), BCa cancerous tissues (r = −0.440, P = 0.001) and peripheral blood cells of healthy controls (r = −0.315, P = 0.035) were observed.

Using one-way ANOVA test, significant differences of LINE-1 methylation levels in blood, urine and cancerous tissue DNAs were not observed among BCa patients with different smoking status (non-smokers, current smokers and quitted smokers) (P = 0.477, 0.711 and 0.964, respectively). Likewise, levels of urinary TAS and plasma protein carbonyl content among the different smoking status were not significantly different (P = 0.053 and 0.093, respectively). Urinary TAS (P = 0.055 for healthy, P = 0.879 for BCa) and plasma protein carbonyl content (P = 0.634 for healthy, P = 0.072 for BCa) were not significantly different between males and females both in healthy and BCa groups. However, positive correlation between urinary TAS and age in healthy group (r = 0.374, P = 0.011) and negative correlation between plasma protein carbonyl content and BMI in BCa group (r = −0.261, P = 0.044) were revealed.

**Figure 5:**
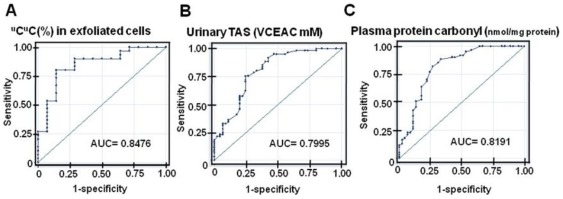
The ROC curves of ^u^C^u^C determination, the urinary TAS and the plasma protein carbonyl content. A: The % ^u^C^u^C in the urinary exfoliated cells. B: The urinary TAS. C: The plasma protein carbonyl content. The determination of ^u^C^u^C in the urinary exfoliated cells had the highest AUC, which suggests the highest diagnostic potential.

A ROC analysis was performed to evaluate how well the determination of the LINE-1 methylation can discriminate between the BCa patients and healthy individuals. Among the various patterns of LINE-1 methylation, the ^u^C^u^C level in the urinary exfoliated cells had the greatest diagnostic value with the highest AUC at 0.848 (95% CI: 0.719–0.977) (Table 1 and Fig. 5A). At a 38.43% cutoff, sensitivity, specificity and accuracy of the urinary ^u^C^u^C determination were 80.00%, 85.00% and 81.82%, respectively. The AUC values of the % methylation in both the blood (0.684) and the urine (0.691) cells were lower than that of urinary ^u^C^u^C (Figure S3). Thus, the determination of ^u^C^u^C in the urinary exfoliated cells provided the highest diagnostic potential relative to the other forms of methylation.

**Table 1:** The ROC analysis and diagnostic values of the measurements of the LINE-1 methylation and ^u^C^u^C levels as well as oxidative stress biomarkers.

Samples	Measurements	AUC	Cutoff	Sensitivity (%)	Specificity (%)	Accuracy (%)
Peripheral blood cells	Methylation (%)	0.684	42.87	96.67	40.00	72.38
	^u^C^u^C (%)	0.641	34.64	50.00	77.78	61.90
Urinary exfoliated cells	Methylation (%)	0.691	42.15	100.00	21.43	75.00
	^u^C^u^C (%)	0.848	38.43	80.00	85.00	81.82
Urine	TAS (VCEAC mM)	0.800	1.32	88.52	60.00	76.42
Plasma	Protein carbonyl	0.820	0.44	81.97	73.33	78.30
	(nMol/mg proteins)					

The ROC curves of the urinary TAS and plasma protein carbonyl content were also generated. The urinary TAS had an AUC of 0.800 (95% CI: 0.711–0.888) and had sensitivity, specificity and accuracy of 88.52%, 60.00%, and 76.42%, respectively, at a cutoff of 1.32 VCEAC mM (Table 1 and Fig. 5B). An AUC value for the plasma protein carbonyl content was 0.819 (95% CI: 0.733–0.905), and its sensitivity, specificity and accuracy, at a cutoff of 0.44 nMol/mg of protein, were 81.97%, 73.33% and 78.30%, respectively (Table 1 and Fig. 5C).

**Figure 6:**
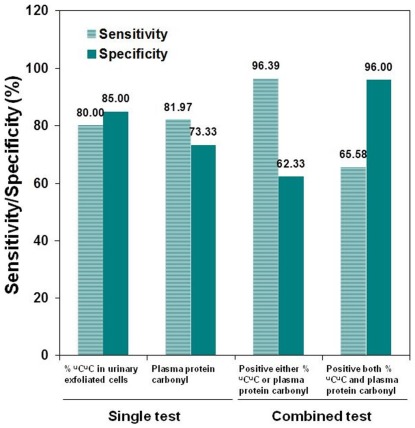
The sensitivity and specificity of the combined test of % ^u^C^u^C in the urinary exfoliated cells and the plasma protein carbonyl content. An increased sensitivity (96.39%) was achieved when either the % ^u^C^u^C in urinary exfoliated cells or the plasma protein carbonyl content was positive. Additionally, an increased specificity (96.00%) of the test was achieved when at least one of both markers was positive.

We further evaluated whether the combination of ^u^C^u^C in the urinary exfoliated cells and the plasma protein carbonyl content would improve the diagnostic potential for BCa. The test criteria revealed that two positive results showed a higher specificity (96.00% with sensitivity of 65.58%) and a higher sensitivity (96.39% with specificity of 62.33%) when at least one marker was positive (Fig. 6).

## Discussion

The loss of DNA methylation in the LINE-1 elements is suggested to be a cardinal event in cancer development because it promotes genomic instability and alters gene expression [12]–[16]. The mechanism that causes this loss of DNA methylation is unknown. The evaluation of the correlation of LINE-1 methylation levels between several loci suggested that the LINE-1 hypomethylation mechanism is generalized [23]. The timing of DNA methylation and bladder cancer is not yet clear. Rather, it has been shown to be a biomarker in post-diagnostic sample analyses, however, this relationship has not been shown in using pre-diagnostic samples, and it is not yet clear whether the lower methylation observed was caused by the carcinogenic process itself [30]. In the present study, we demonstrated here for the first time a significant positive correlation between LINE-1 hypomethylation and oxidative stress not only in cancer patients but also in healthy individuals. Therefore, oxidative stress may be one of the causes or consequences of LINE-1 hypomethylation.

Currently, to our knowledge, there is no biochemical mechanism that implies how LINE-1 hypomethylation can produce ROS. However, the mechanism of how oxidative stress reduces DNA methylation has been proposed [31], and the oxidative stress-induced DNA methylation change appears to be temporal [32], [33]. Oxidized DNA lesions that are induced by ROS, such as 8-OHdG (in CpG dinucleotides), can strongly inhibit the methylation by a DNA methyltransferase at the adjacent C residues [34]. Additionally, an unfixed 8-OHdG may introduce a G-T transversion resulting in the loss of CpG dinucleotides [35]. Additionally, in an oxidative stress condition, the resynthesis of glutathione (GSH) in response to GSH depletion through the methionine cycle in one-carbon metabolism pathway is increased. This pathway requires *S*-adenosylmethionine (SAM) for synthesizing the homocysteine to be used for the GSH synthesis, leading to a decreased availability of SAM for DNA methylation [36]. The agents and conditions that induce GSH depletion have been demonstrated to impair DNA methylation [37], [38]. Therefore, oxidative stress is thought to alter the methylation of DNA, which leads to changes in the gene expression that could contribute to tumor development [7]. In addition to decreased availability of SAM, depletion of methyl pool in folate-deficient models has been shown to cause DNA hypomethylation [39], [40].

The LINE-1 methylation levels have been studied in several sources of DNA to improve cancer diagnosis. Unfortunately, there are tissue specific methylation levels and that these differ depending upon the methylation biomarker used to measure methylation [15]. In clinical specimens both cancerous and normal cells are usually coexisted, which means that based only on the LINE-1 methylation levels cancer DNA cannot be effectively detected when the DNA sources are contaminated with DNA from various types of normal cells. For detection of noninvasive BCa by DNA examination, blood and urine are the common sources of DNA. Although cancer cells are present in both sources, the contamination with various types of normal cells is usually inevitable. Previously, we tested the DNA from both an oral rinse and from the white blood cells of patients with oral cancer and found that the percentage of hypomethylated loci or ^u^C^u^C was more specific to the cancer DNA than the overall LINE-1 methylation levels [26], [41]. Moreover, the LINE-1 methylation levels between the cell types (oral epithelial and blood cells) were different because of the number of partial LINE-1 methylation loci. Here, we showed similar data in BCa; the number of LINE-1 hypomethylated loci in urinary exfoliated cells is a better tumor marker than the overall LINE-1 methylation level.

The increase in oxidative stress in BCa is well recognized and is believed to be critically involved in urothelial carcinogenesis [3]–[6]. The study results support this association; there still need to be experiments or analyses conducted using pre-diagnostic samples to confirm the temporality of the association observed here. The plasma protein carbonyl content was increased in the patients with BCa relative to the levels in the healthy controls [42]. The total antioxidant activity measured in the plasma of urothelial bladder carcinoma patients was lower than that in healthy controls [43]. In this study, we used the urinary TAS to reflect the overall antioxidant capacity in the body because the urinary TAS is a well-accepted marker for estimating the total antioxidants in biological fluids [44]. Based on our findings, we conclude that the patients with BCa had an increase in oxidative stress. When combined with the LINE-1 hypomethylation levels, we hypothesize that the deprivation of antioxidants or an increase in oxidative stress may influence the global hypomethylation that promotes carcinogenesis.

The ROC analysis revealed the promising diagnostic potential of the measurements for the plasma protein carbonyl content and the LINE-1 hypomethylated loci in urinary exfoliated cells. The combination of these two tests increased the sensitivity and specificity of the diagnostic values to 96%. This association needs to be evaluated in pre-diagnostic samples. To date, there is no biomarker that can replace the need for cystoscopy, and the discovery of a new BCa biomarker is still a challenge for the field [45]. Although many urinary biomarkers have been reported to detect BCa with high sensitivity, their specificity is much lower than the urine cytology (95–100%) [45], [46]. A meta-analysis reported that the sensitivity of urine cytology is only 34% [47]. Thus, a novel, noninvasive test with a high sensitivity and specificity that is comparable to that of urine cytology is required. Relative to the previously reported biomarkers for detecting BCa [45]–[48], the current combination tests have shown to have a diagnostic potential in the pre-diagnostic samples.

In conclusion, we found a positive correlation between the LINE-1 hypomethylation of urine exfoliative cells and the oxidative stress in both normal individuals and BCa patients. ROS is hypothesized to be one of the candidate mechanisms that can reduce LINE-1 methylation. Moreover, the ROS generation and LINE-1 methylation levels and patterns in the blood, urinary exfoliated cells and cancer cells from patients were significantly changed relative to healthy controls. The combined information from these two tests demonstrated a very high sensitivity and specificity for BCa diagnosis, however, it needs to be confirmed using pre-diagnostic samples, prior to implementation in cancer diagnosis. Further studies of global hypomethylation and ROS will not only improve our knowledge of carcinogenic mechanisms but will also improve our management of the disease.

### Statement of translational relevance

Patients with bladder cancer have increased oxidative stress and LINE-1 hypomethylation relative to healthy controls. LINE-1 hypomethylation is correlated with an increased ROS generation in both normal and cancer subjects, indicating a reverse association between LINE1 methylation and oxidative stress. LINE-1 hypomethylation is known to promote genome instability, alter gene expression and contribute to tumor progression. Thus, the treatment that effectively reduces the oxidative stress in the patients may attenuate the LINE-1 hypomethylation and decelerate the progression of the tumor. Until now, there has been no noninvasive biomarker for detecting a bladder tumor that can replace the need for cystoscopy. Based on pre-diagnostic samples, the determination of the hypomethylated loci of LINE-1 in urinary exfoliated cells combined with the plasma protein carbonyl content show high sensitivity and specificity for bladder cancer prediction. This combination test may be clinically useful for bladder cancer diagnosis and monitoring of treatment thus reducing the frequency of invasive cystoscopic procedures. However, the test should be performed in pre-diagnostic samples before the actual diagnostic power of the test can be evaluated.

## Supporting Information

Figure S1 **Comparison of partial methylation loci of LINE-1 in blood and urinary exfoliated cells as well as cancerous tissues of bladder cancer patients and healthy controls.**
(DOC)

Figure S2 **Correlations between urinary TAS and LINE-1 methylation patterns (^m^C^m^C, ^u^C^u^C, ^u^C^m^C and ^m^C^u^C) in peripheral blood cells (A–D and I–L) and cancerous tissues (E–H) of the patients with BCa (A–H) and healthy controls (I–L).**
(DOC)

Figure S3 **ROC curves of various forms of LINE-1 methylation in blood and urine cells.**
(DOC)
