# Supplementary material for: Long Interspersed Nuclear Element-1 Hypomethylation and Oxidative Stress: Correlation and Bladder Cancer Diagnostic Potential
Source: PLoS One. 2012 May 15;7(5):e37009. doi: 10.1371/journal.pone.0037009 (PMC3352860; doi:10.1371/journal.pone.0037009)

**Figure S1** Comparison of partial methylation loci of LINE-1 in blood and urinary exfoliated cells as well as cancerous tissues of bladder cancer patients and healthy controls.


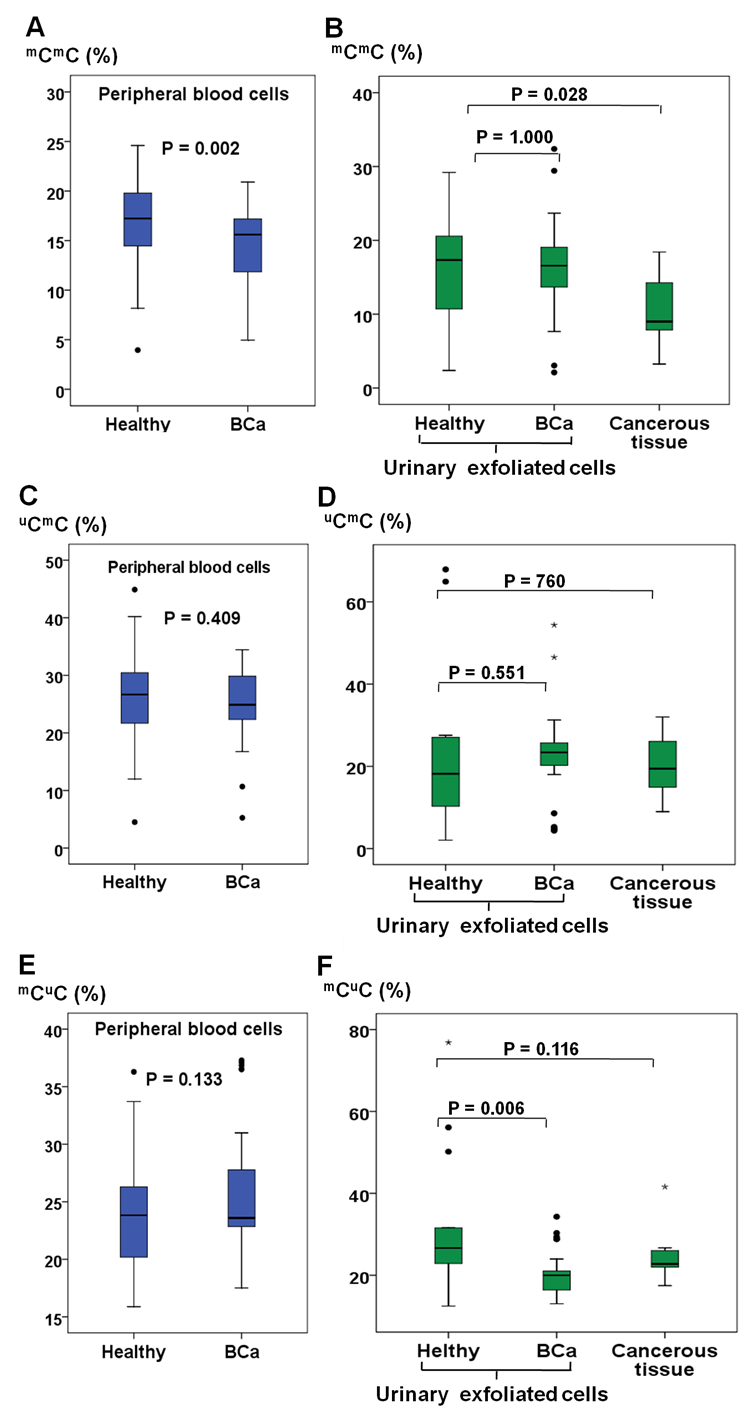

Supplement: Figure S1 — Comparison of partial methylation loci of LINE-1 in blood and urinary exfoliated cells as well as cancerous tissues of bladder cancer patients and healthy controls. (DOC) [file pone.0037009.s001.doc]
