# Supplementary material for: Long Interspersed Nuclear Element-1 Hypomethylation and Oxidative Stress: Correlation and Bladder Cancer Diagnostic Potential
Source: PLoS One. 2012 May 15;7(5):e37009. doi: 10.1371/journal.pone.0037009 (PMC3352860; doi:10.1371/journal.pone.0037009)

**Figure S2** Correlations between urinary TAS and LINE-1 methylation patterns (^m^C^m^C, ^u^C^u^C, ^u^C^m^C and ^m^C^u^C) in peripheral blood cells (A-D and I-L) and cancerous tissues (E-H) of the patients with BCa (A-H) and healthy controls (I-L).


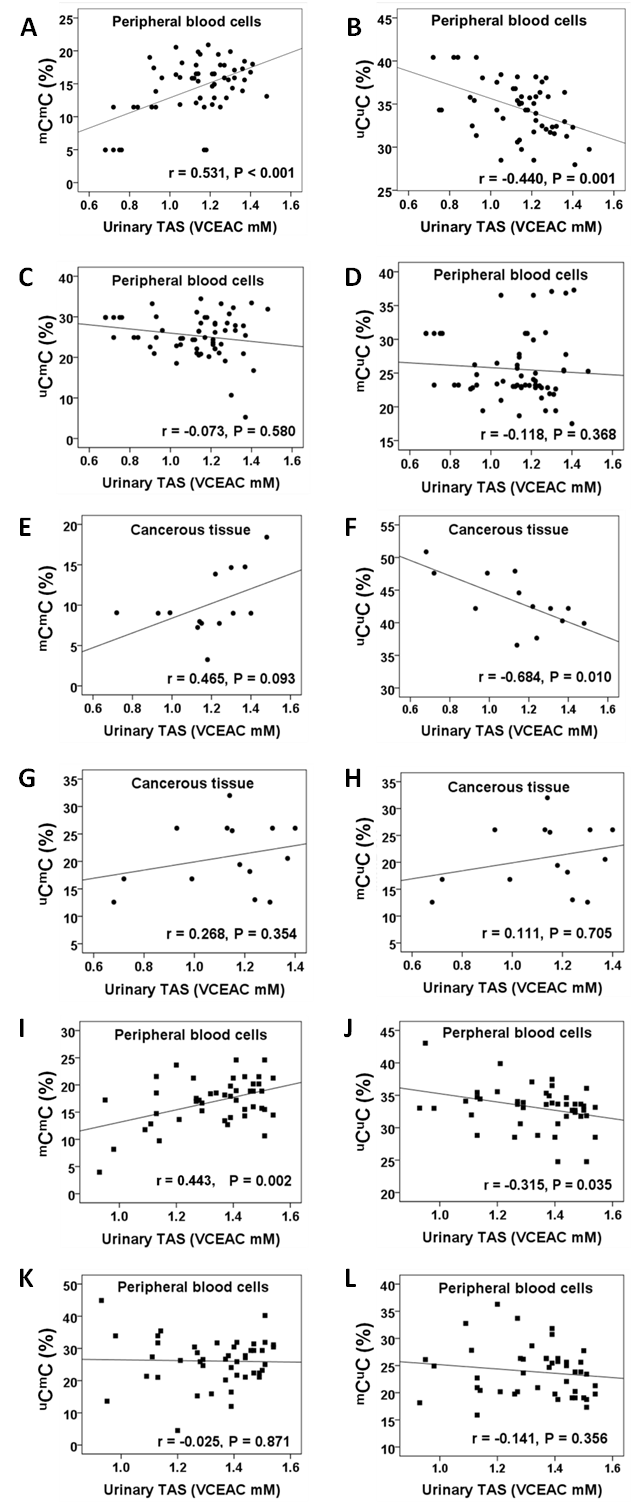

Supplement: Figure S2 — Correlations between urinary TAS and LINE-1 methylation patterns (mCmC, uCuC, uCmC and mCuC) in peripheral blood cells (A–D and I–L) and cancerous tissues (E–H) of the patients with BCa (A–H) and healthy controls (I–L). (DOC) [file pone.0037009.s002.doc]
