# Supplementary figures and images for: Long Interspersed Nuclear Element-1 Hypomethylation and Oxidative Stress: Correlation and Bladder Cancer Diagnostic Potential
Source: PLoS One. 2012 May 15;7(5):e37009. doi: 10.1371/journal.pone.0037009 (PMC3352860; doi:10.1371/journal.pone.0037009)

**Figure S3** ROC curves of various forms of LINE-1 methylation in blood and urine cells.


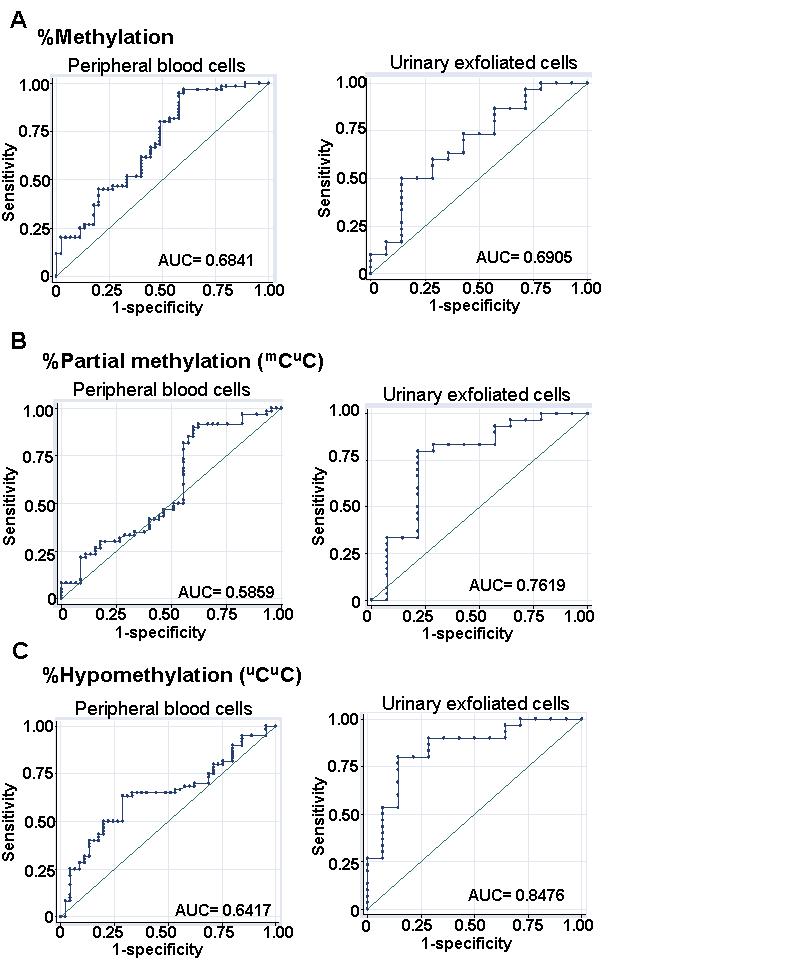

Supplement: Figure S3 — ROC curves of various forms of LINE-1 methylation in blood and urine cells. (DOC) [file pone.0037009.s003.doc]
